# Antibacterial Potential and Biocompatibility of Chitosan/Polycaprolactone Nanofibrous Membranes Incorporated with Silver Nanoparticles

**DOI:** 10.3390/polym16121729

**Published:** 2024-06-18

**Authors:** Viktoriia Korniienko, Yevgeniia Husak, Kateryna Diedkova, Yuliia Varava, Vladlens Grebnevs, Oksana Pogorielova, Māris Bērtiņš, Valeriia Korniienko, Baiba Zandersone, Almira Ramanaviciene, Arunas Ramanavicius, Maksym Pogorielov

**Affiliations:** 1 Institute of Atomic Physics and Spectroscopy, University of Latvia, Jelgavas iela 3, LV-1004 Riga, Latvia; kateryna.diedkova@lu.lv (K.D.); baiba.zandersone@lu.lv (B.Z.); maksym.pogorielov@lu.lv (M.P.); 2 Biomedical Research Centre, Sumy State University, R-Korsakova Street, 40007 Sumy, Ukraine; yevheniia.husak@polsl.pl (Y.H.); yuliia.varava@gmail.com (Y.V.); o.pogorelova@med.sumdu.edu.ua (O.P.); v.kornienko@med.sumdu.edu.ua (V.K.); 3 Faculty of Chemistry, Silesian University of Technology, 44-100 Gliwice, Poland; vladlens.grebnevs@lu.lv; 4 Faculty of Chemistry, University of Latvia, Jelgavas iela 1, LV-1004 Riga, Latvia; maris.bertins@lu.lv; 5 NanoTechnas-Center of Nanotechnology and Materials Science, Institute of Chemistry, Faculty of Chemistry and Geosciences, Vilnius University, Naugarduko Str. 24, LT-03225 Vilnius, Lithuania; almira.ramanaviciene@chf.vu.lt

**Keywords:** chitosan, polycaprolactone (PCL), electrospun nanofibers, silver nanoparticles (AgNPs), antibacterial potential, antimicrobial efficiency, biocompatibility, electrospinning, Gram-negative bacteria, Gram-positive bacteria

## Abstract

This study addresses the need for enhanced antimicrobial properties of electrospun membranes, either through surface modifications or the incorporation of antimicrobial agents, which are crucial for improved clinical outcomes. In this context, chitosan—a biopolymer lauded for its biocompatibility and extracellular matrix-mimicking properties—emerges as an excellent candidate for tissue regeneration. However, fabricating chitosan nanofibers via electrospinning often challenges the preservation of their structural integrity. This research innovatively develops a chitosan/polycaprolactone (CH/PCL) composite nanofibrous membrane by employing a layer-by-layer electrospinning technique, enhanced with silver nanoparticles (AgNPs) synthesized through a wet chemical process. The antibacterial efficacy, adhesive properties, and cytotoxicity of electrospun chitosan membranes were evaluated, while also analyzing their hydrophilicity and nanofibrous structure using SEM. The resulting CH/PCL-AgNPs composite membranes retain a porous framework, achieve balanced hydrophilicity, display commendable biocompatibility, and exert broad-spectrum antibacterial activity against both Gram-negative and Gram-positive bacteria, with their efficacy correlating to the AgNP concentration. Furthermore, our data suggest that the antimicrobial efficiency of these membranes is influenced by the timed release of silver ions during the incubation period. Membranes incorporated starting with AgNPs at a concentration of 50 µg/mL effectively suppressed the growth of both microorganisms during the early stages up to 8 h of incubation. These insights underscore the potential of the developed electrospun composite membranes, with their superior antibacterial qualities, to serve as innovative solutions in the field of tissue engineering.

## 1. Introduction

Regenerative therapy is predicated on the modulation of cellular and molecular processes that underlie the repair or replacement of injured or degenerated tissues, ultimately aiming to reinstate both the function and structure of complex tissue architectures. In the realm of biomedicine, biomaterials such as agarose, alginate, chitosan, fibrin, and collagen are widely utilized within tissue engineering strategies due to their facilitative properties in cellular support and tissue integration. Advancements in nanotechnology have opened a new era of nanomaterials, including nanofibers, nanotubes, and nanoparticles, which demonstrate enhanced outcomes when compared with conventional grafting methods [1,2]. These outcomes can be attributed to their exceptional mechanical properties, highly porous configuration, improved biocompatibility, and tunable biodegradability. Polymer-based scaffolds at the nanoscale level are particularly advantageous as they offer a platform for direct cellular interaction with nanostructured extracellular matrices (ECM), thereby promoting cellular adhesion, proliferation, and differentiation, which are critical to efficacious tissue regeneration [3].

Among other methods, electrospinning is a highly efficient technique for producing nanofibers, typically with average diameters ranging from several tens to hundreds of nanometers. This method proves effective in minimizing processing-related factors, addressing limitations at the molecular structure level, and enhancing the surface properties of materials. As a result, electrospinning improves the biological tissue compatibility of polymers and provides a superior effect in regenerative medicine [4].

Polycaprolactone (PCL) and chitosan (CH) are widely recognized in the field of biomaterials for their excellent biocompatibility and biodegradability, which render them highly suitable for a multitude of biomedical applications, particularly within the realms of medicine and tissue engineering. PCL, in particular, has emerged as a frontrunner for medical use due to its optimal blend of chemical, mechanical, and biological features. The structural mimicry of PCL nanofibers to the ECM is noteworthy, offering a large specific surface area, elevated porosity, fine pore size, and suitable mechanical attributes [5]. These traits make PCL nanofibers exceedingly conducive to medical utilization. Furthermore, the inherent porosity of electrospun fibers augments the versatility of these materials, enabling effective drug encapsulation and release mechanisms. This, coupled with their physiochemical properties, underpins the growing interest in PCL and CH polymers as pivotal components in the development of innovative tissue engineering solutions [6].

Chitosan has a carbohydrate backbone structure similar to cellulose, consisting of two types of repeating units: N-acetyl-D-glucosamine and D-glucosamine, linked by (1 → 4)-β-glycosidic bonds. Chitosan electrospun biomaterials are distinguished by their expansive specific surface area and porosity, characterized by a hierarchical pore size distribution [7]. Due to their notable biocompatibility, biodegradability, inherent antibacterial properties, and anti-inflammatory capabilities, electrospun CH fibers are promising candidates for applications in tissue engineering and regenerative medicine. Chitosan-based nanocomposites, owing to their versatility, can be engineered into scaffolds of varying forms and dimensions, adeptly mimicking the natural extracellular matrix and thus facilitating cell adhesion, proliferation, and differentiation—critical processes for creating an ideal microenvironment for tissue regeneration [8].

In the context of guided tissue regeneration (GTR) and bone regeneration (GBR) therapies, barrier membranes play a pivotal role in tissue compartmentalization. There is a growing preference for resorbable membranes because of their superior characteristics and their ability to obviate the need for secondary surgery. Nonetheless, the exposure of these membranes can precipitate microbial contamination, thereby undermining their integrity and compromising the results of regeneration efforts. The clinical impact of microbial infections on regenerative outcomes is profound, yet there remains a paucity of clinical research, particularly concerning the colonization patterns of diverse membrane types. A deeper understanding of biofilm formation on exposed membranes is imperative for elucidating the mechanisms underlying impaired tissue regeneration [9].

The risk of infection in tandem with regenerative procedures is escalating in proportion to their increased prevalence [10]. Consequently, the development of customized antimicrobial protocols for the use of membranes, grafts, and implants becomes crucial. Such guidelines are fundamental to establishing a rigorous framework for infection control within regenerative therapies, underscoring the necessity for comprehensive management strategies to mitigate infection risks.

Electrospinning emerges as a quintessential fabrication technique for engineering biomaterials that exhibit tailored structural, mechanical, and biological attributes. This versatile modality is particularly instrumental in generating nanofibrous matrices, which are indispensable in the domain of tissue engineering and regenerative medicine [11]. Through the strategic amalgamation of materials such as PCL and CH with biocompatible polymers, it is feasible to synthesize effective scaffolds that exhibit enhanced biocompatibility for various biomedical applications. The integration of nanoparticles within these scaffolds significantly fortifies their structural framework while also facilitating a sustained drug release [12]. Such a controlled release mechanism is invaluable across a spectrum of medical and therapeutic contexts. Specifically, electrospun nanofibers composed of CH and PCL, when impregnated with silver nanoparticles, are shown to effectuate the discharge of Ag+ ions in a concentration deemed sufficient for antimicrobial activity [13].

In our previous work, we synthesized a range of electrospun membranes composed of polylactic acid (PLA), PCL, and CH, exhibiting high biocompatibility levels, desirable biodegradation profiles, and anti-inflammatory properties [14,15]. However, the CH-based membranes lacked sufficient antibacterial efficacy, necessitating the inclusion of additional components to satisfy antimicrobial standards [14]. A further limitation associated with CH membranes was their accelerated degradation rate in aqueous environments, which prompted the need for enhanced support structures or effective crosslinking strategies [16]. Although silver nanoparticles (AgNPs) are recognized for their potent antibacterial properties, particularly against multi-drug resistant strains such as MRSA, their biocompatibility remains suboptimal, necessitating further optimization to mitigate adverse cellular and tissue interactions. Our prior investigations have shown that AgNPs synthesized within a polyvinylpyrrolidone matrix and subsequently purified using a reverse osmosis membrane exhibit both high antibacterial potency and acceptable biocompatibility [15].

While a myriad of such scaffolds are subject to ongoing investigative scrutiny through in vitro and in vivo studies to ascertain their proficiency in tissue repair, it is pivotal to recognize that the ingenious fusion of biocompatible polymers with antibacterial agents like silver nanoparticles harbors the promise for the advent of exceptionally efficacious scaffolds. These advancements, via electrospinning technologies, signify a progressive leap in tissue regeneration methodologies [17].

Building upon these findings, the present study focuses on developing a novel chitosan/polycaprolactone (Ch/PCL) layer-by-layer membrane, incorporating AgNPs, and evaluating its structural integrity and biological response.

## 2. Materials and Methods

### 2.1. Materials

Polycaprolactone (PCL), Mn = 80,000 g/mol, Chitosan powder (890,000 Da, Glentham Life Sciences in Corsham, UK, CAS 9012-76-4) and Poly(ethylene oxide) (PEO, MW 1500, CAS 25322-68-3) were obtained from Sigma Aldrich (Saint Louis, MI, USA). Chloroform (purity ≥ 99%) was purchased from Chempur (Piekary Śląskie, Poland), and N, N-Dimethylformamide (DMF) and Acetic Acid (AA, CAS 7732-18-5, purity ≥ 99.8–100.5%) were derived from Honeywell (Charlotte, NC, USA).

### 2.2. Preparation of Ch/PEO Solution

To create a CH/PEO solution, 0.04 g of Ch powder and 0.18 g of PEO were dissolved in 8 mL of 50% of aqueous AA by 24 h stirring with 300 rpm on a magnetic stirrer at room temperature until a uniform solution was achieved. Before electrospinning, the solutions were allowed to rest at room temperature for 30 min to eliminate any trapped air bubbles.

### 2.3. Preparation of Polycaprolactone Solution

To prepare the electrospinning solution, a total of 0.96 g of PCL was completely dissolved in 8 mL of a solvent mixture consisting of chloroform and DMF, in a ratio of 3:1. The dissolution process involved stirring the mixture at a speed of 300 rpm on a magnetic stirrer for 3 h, at room temperature.

### 2.4. Electrospinning of CH/PCL Nanofiber Membranes

A layer-by-layer electrospinning method was used to obtain Ch/PCL electrospun membranes, where the PCL nanofiber membrane was used as a basis. For the production of PCL electrospun fiber mats, we utilized the following parameters: a power of 17 kv, a distance of 170 mm from the tip to the collector, a feeding rate of 1 mL per hour, and a rotation speed of the drum (with a diameter of 7 cm) set to 350 rpm.

A CH/PEO membrane was spun on top of the PCL electrospun scaffold using the following parameters: a feeding rate of 200 µL per hour, a power of 17 kv, a distance of 13 cm from the tip to the collector, a needle inner diameter of 0.8 mm, and a rotation speed of the drum set to 400 rpm.

### 2.5. Neutralization Treatments of Chitosan Nanofiber Membranes

To prevent the chitosan nanofiber membranes from dissolving in the aqueous medium, they were neutralized in a Na-based solution [14]. Briefly, the treatment was performed by neutralizing each mat in 1 M NaOH (aqueous or 70% ethanol/30% water solution) in a 24-well plastic plate for 12 h. After the immersion, the membranes were washed repeatedly with distilled water and dried at ambient conditions for 1 day at room temperature.

### 2.6. Functionalization of CH/PCL Nanofiber Membranes with AgNPs

Treated electrospun nanofibers were exposed to functionalization with the silver nanoparticles (Ag NPs) supplied by Nano Pure Co., Warsaw, Poland. AgNO_3_, PVP, and NaClO were combined in borosilicate bottles. The reaction took place in an aqueous solution, using light from commercially available 1 W light-emitting diodes (λ = 420 nm) as the catalyst. A detailed description of the synthesis of AgNPs-2, along with their structural and chemical parameters, can be found in [18]. The AgNPs utilized in the experiment exhibit a spherical morphology, with an average size of 27 ± 4.3 nm [13]. AgNPs were added to the Ch/PCL membrane by drop-coating with the following concentrations: 12.5 µg/mL, 25 µg/mL, 50 µg/mL, 100 µg/mL, 200 µg/mL, and 400 µg/mL. Subsequent samples were dried for 24 h at room temperature.

### 2.7. Scanning Electron Microscopy (SEM) with EDX, Measurement of Contact Angle

#### 2.7.1. Scanning Electron Microscopy

Samples 5 × 5 mm from each electrospun membrane were observed using scanning electron microscopy (SEO-SEM Inspect S50-B, SELMI, Sumy, Ukraine; Phenom ProX, Phenom-World BV, Eindhoven, The Netherlands), which was equipped with an energy-dispersive X-ray spectrometer (EDX). Fiber morphologies were analyzed based on the scanning electron microscopy micrographs. Fiber diameter and ‘porous area fraction’ were measured using Fiji software (ImageJ 1.51f; Java 1.8.0_102) [19]. ‘Porous area fraction’ was detected with computer binary image analysis. The determination involved categorizing images into two categories: black areas representing pores and white areas representing the substrate, achieved through gray-level thresholding. The ‘porous area fraction’ was calculated as the ratio of pore area to the total area within the image region under examination. Fiber diameter distribution frequency histograms were constructed using Excel software V 16.84 (Office 365 ProPlus). Fibers were randomly chosen from three electrospun membranes of each sample type (100 fibers from each specimen). The fiber diameters and ‘porous area fraction’ are reported as average values with standard deviation.

The ImageJ software judged the fiber orientation on the basis of a color-coded map. The angle of local orientation can range from −90° to −90° relative to the horizontal.

#### 2.7.2. Cross-Section with EDX Membranes

The cross-section of the membranes was obtained by cutting the samples. The cross-section view and its chemical composition were observed by SEM and the EDX line scan. The samples were fixed by an EM-Tec S-Clip sample holder with a 1xS-Clip (Micro to Nano BV, Haarlem, Netherlands) at 90° to an electron beam. The copper sample clip held a thin membrane on a holder and allowed for the micrographs to be obtained without the need for conductive adhesives or conductive pastes.

#### 2.7.3. 3D Visualization of Membranes

Three-dimensional images and submicrometer roughness measurements of the samples were generated by the Phenom desktop scanning electron microscope. Based on “shape from shading” technology, 3D images were obtained and used to interpret the sample’s structure. The roughness characteristics were presented via the average roughness (Ra) and the roughness height (Rz).

#### 2.7.4. Measurement of Contact Angle

The membrane surface’s wettability was measured with 2 µL of deionized water droplets using the video-based optical contact angle measuring equipment OCA 15 EC, Series GM-10-473 V-5.0 (Data Physics, Filderstadt, Germany). Static contact angles were used to evaluate the hydrophobicity/hydrophilicity of a solid surface—the values of the static contact angles we were using on the surface. The camera fixes at the moment of the first interaction between the water and the surface of the membrane, and it is expressed as the constant contact angle and, accordingly, the instant wetting characteristics of the substrate. The wettability values were measured on three different membranes, and an average of three readings was reported for each sample.

### 2.8. Fourier Transform Infrared Spectroscopy (FTIR)

Fourier transform infrared (FTIR) spectra of the compounds were captured using the Perkin Elmer Frontier instrument (Waltham, MA, USA) in the ATR mode, and the following spectra recording parameters were used: wavenumber range, 3100 to 600 cm^−1^; resolution, 4 cm^−1^; number of scans, 64.

### 2.9. Release of Silver Ions and ICP-MS Analysis

Previously prepared CH/PCL nanofiber membranes, functionalized with AgNPs (Section 2.6), were immersed in 50 mL of deionized water (grade 1, EC < 0.055 uS/cm) for the release studies. Samples from this solution were collected at regular intervals of 1, 3, 6, 14, and 24 h. The Ag concentration in the collected samples was measured using ICP-MS.

To prepare the samples for analysis, each sample underwent acidification using high-purity nitric acid (TraceMetal™ Grade, Fisher Chemical™, Sigma Aldrich (Saint Louis, MI, USA)) until reaching a final concentration of 2% (*v*/*v*). Subsequently, the acidified samples were introduced into the Agilent 8900 ICP-MS QQQ instrument (Waltham, MA, USA), which was equipped with a micro-mist nebulizer and a He collision cell for analysis. The instrumental parameters for the ICP-MS analysis were established and are summarized in Table 1.

To ensure accuracy, blank correction was employed, and ultraclean reagents (TraceMetal™ Grade, Fisher Chemical™) along with suitable blanks were utilized to monitor and correct any potential background contamination. The system’s stability during measurements was controlled using an internal standard solution (10 µg L^−1^, Agilent, Waltham, MA, USA)). Additionally, for every ten samples, two standard solutions (10 µg L^−1^) were analyzed to confirm the stability of the system and validate the accuracy of the results.

Data processing, collection, and result calculations were performed using the MassHunter workstation program, version 12, which included the subprograms for instrument control and offline data analysis.

### 2.10. Antibacterial Activity Study

*E. coli* (ATCC 25922) and *S. aureus* (ATCC 25923) were selected to evaluate the antibacterial activity of the CH/PCL membranes. Strains were cultured in nutrient broth at 37 °C for 24 h. Samples of 0.5 cm^2^ (for bacterial growth-viability assay and biofilm formation study) and disks of 5 mm in diameter (for disk-diffusion assay) were sterilized with UV light radiation at 254 for 15 min on each side.

#### 2.10.1. Kirby–Bauer Disk-Diffusion Method

Petri plates were inoculated by swabbing bacterial broth (105 CFU/mL) evenly spread on a solid medium plate. Subsequently, nanofibrous membrane disks were positioned on the plate, and the plates were inverted before being incubated at 37 °C. After 24 h, the inhibition zone diameter, encompassing the disk diameter, was measured.

#### 2.10.2. Time-Dependent Bacterial Growth Assay

Membrane samples were placed in a sterile 24-well plastic plate with 2 mL of pre-prepared bacterial suspension (1 × 10^5^ CFU/mL). Bacteria suspended in nutrient broth were used as a control. After incubation for 2, 4, 6, and 8 h, aliquots of 200 μL from each well were transferred to a 96-well microtitre plate. The degree of opacity was assessed by measuring the medium’s transmittance using a microplate reader (Multiskan FC, Thermo Fisher Scientific, Waltham, MA, USA) at wavelengths of 595 nm [14]. The turbidity observed in microbial growth without the presence of the membrane served as the positive control, while the negative control wells contained a sterile nutrient broth devoid of microbes. Average absorbance values were computed for each set of triplicate controls and samples.

#### 2.10.3. Biofilm Formation Study

The samples were incubated in bacterial broth according to a procedure described for the time-dependent bacterial growth assay (Chapter 2.10.2). After being incubated at 37 °C for 2, 4, 6, and 24 h, the disks underwent three washes with sterile sodium chloride solution to eliminate the non-adherent cells. Subsequently, an ultrasonic bath (model B3500S-MT, Branson Ultrasonics Co., Shanghai, China) was employed to sonicate the samples in tubes containing 1.0 mL of sterile saline solution for 1 min to remove the bacteria adhered to the specimen surfaces. Following this step, 10 μL aliquots of saline solution were inoculated onto nutrient agar plates using the streak-plating technique to quantify the bacterial count after 24 h of incubation at 37 °C. Control samples, consisting of growth medium without bacterial inoculum, were also included in the experiment.

Following a 24 h incubation in a bacterial suspension, an additional set of the samples underwent preparation for SEM study. This aimed to assess the attachment, dissemination, and morphology of bacterial cells on CH nanofibers using a procedure that has been previously described [16].

### 2.11. Biocompatibility Assessment

The human keratinocytes (HaCaT) (CLS Cell Lines Service^®^, Eppelheim, Germany) 1q with 54 passages (p54) were used for the assessment of cytotoxicity and biocompatibility of the samples. The materials were UV sterilized for 15 min for each side, with the distance between membranes and the UV lamp being 15 cm. Before the experiment, HaCaT cells were grown in 75 cm^2^ cell culture flasks under standard culture conditions of humidified air containing 5% CO_2_ at 37 °C with a medium renewal every 2–3 days. Dulbecco’s modified Eagle medium (DMEM; Sigma-Aldrich, Saint Louis, MO, USA) supplemented with 10% fetal bovine serum (FBS; Sigma-Aldrich, Saint Louis, MO, USA), 100 U/mL of penicillin, and 100 μg/mL of streptomycin (Gibco, Grand Island, NY, USA. The circular samples Ø 5 mm were placed on a 96-well plate (, Numbrecht, Germany, and human keratinocytes were seeded on membranes at a density of 5000 cells/cm^2^ with the addition of complete cell culture media. Cells seeded into wells without membranes served as a positive control, and the medium without cells and membranes was a negative control. The resazurin reduction assay assessed the cell adhesion at 24 h and cell proliferation on the fifth and seventh days. Resazurin (Sigma-Aldrich, Saint Louis, MO, USA) was added to each well, equal to 10% of the medium volume. The resazurin solution was also added to the negative and positive controls. The plates were incubated for 6 h at 37 °C. The absorbance was measured using a Tecan Infinite M200 Pro microplate reader (Tecan Trading AG, Zürich, Switzerland) at 570 and 600 nm wavelengths. The cells were quantified at different time intervals: 1, 5, and 7 days. For each sample, there were triple repetitions.

### 2.12. Statistical Data Processing

Analysis was performed using SPSS software version 8.0 (SPSS Inc., Chicago, IL, USA). All experiments were conducted in triplicate. The results are expressed as mean ± standard deviation. The one-way analysis of variance was used to determine the significance level (*p* < 0.05 was defined as significant).

## 3. Results

### 3.1. Structural Features and Wettability

**Figure 1:**
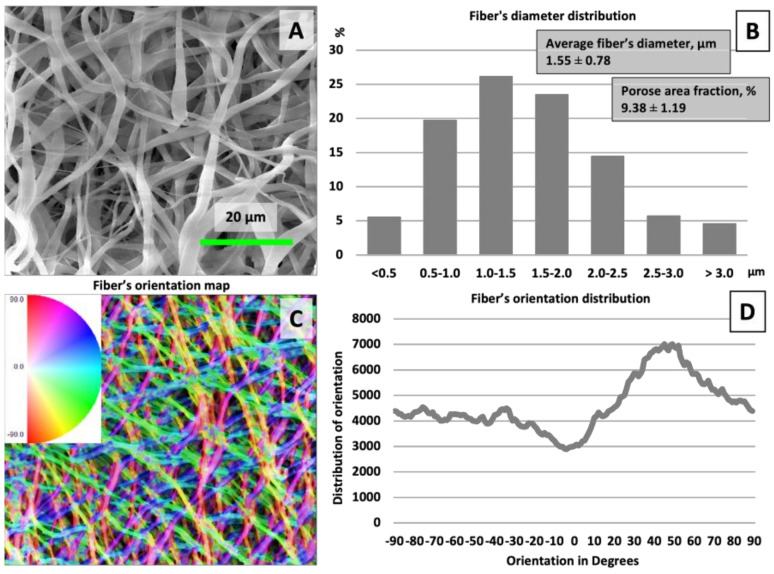
Scanning electron microscopy images of electrospun membranes after alkali treatment (**A**) with a frequency of “fiber’s diameter distribution”, (**B**) “fiber’s orientation map”, and (**C**) “fiber’s orientation distribution” (**D**).

Employing electrospinning as a ‘top-down’ membrane fabrication technique, we created a three-dimensional structure of fibrous mesh with fiber diameters ranging from 0.5 to 3 μm, as illustrated in Figure 1. The fibers were characterized by an average diameter of 1.55 ± 0.78 μm. Optimizing the chemical composition of the solution and the electrospinning parameters enabled the production of uniform, beadless fibers, with a minimal presence of thinner fibers, indicative of the process’s control and precision [20]. The resultant membranes exhibited a porosity of 9.38 ± 1.19%, with fibers predominantly oriented in a random fashion [21]. A predominant fiber alignment was observed at a 50-degree angle despite the random orientation, suggesting an underlying directional tendency in the electrospun nanofibers.

**Figure 2:**
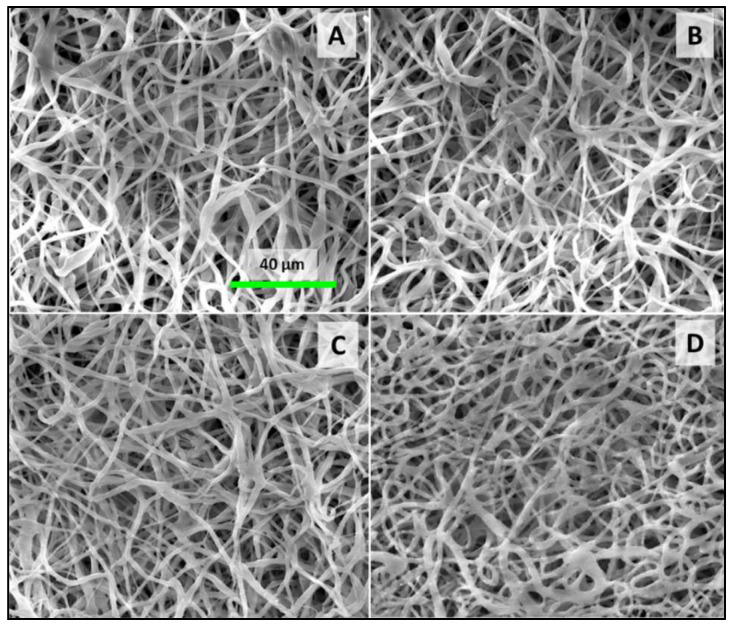
SEM image of membranes loaded with 50 (**A**), 100 (**B**), 200 (**C**), and 400 (**D**) µg of AgNPs.

The morphological examination, as indicated in Figure 2, determined that the incorporation of silver nanoparticles did not alter the morphological characteristics of the fibers; the fiber diameter remained consistent post-addition. SEM analyses confirmed that the samples exhibited a complex structured morphology. Additionally, the porosity of the membranes was maintained across all samples. Such porosity, coupled with the presence of interconnected pores, is anticipated to enhance nutrient and oxygen diffusion, which could be beneficial for subsequent cell culture studies [22].

**Figure 3:**
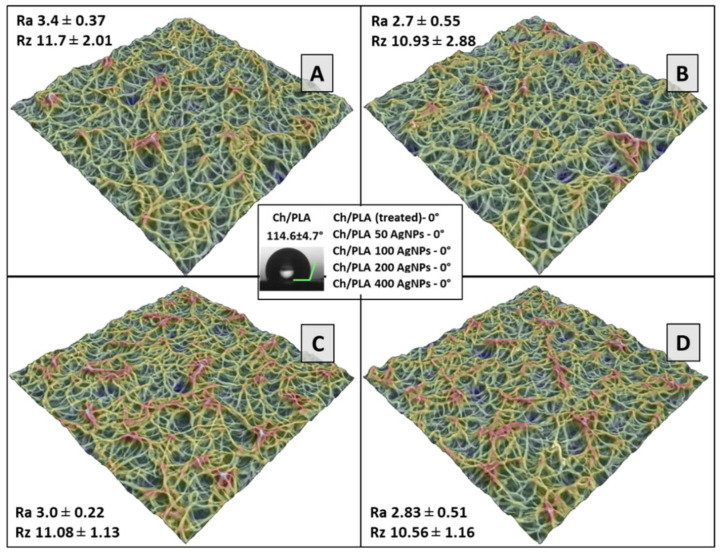
3D reconstruction of surface morphology of electrospun membranes loaded with 50 (**A**), 100 (**B**), 200 (**C**), and 400 (**D**) µg of AgNPs. In the insertions—the roughness reconstruction of the membrane texture (Ra, µm; and Rz, µm). In the centrum—values of the static contact angle measurements.

The three-dimensional (3D) models of the mats, depicted in Figure 3, were instrumental in assessing the membrane morphology. The 3D renderings distinctly showcased the intricate structural composition of the membranes. Quantitative analysis of the surface roughness metrics indicated that there were no statistically significant alterations in the surface morphology attributable to the incorporation of silver.

For membranes to function optimally in wound dressing applications, the inner layer that contacts the skin must exhibit hydrophilic properties to effectively absorb the excess exudate produced by wounds [23,24,25]. Wettability, which is influenced by surface chemistry and morphology, can be modulated through various methods, including the introduction of hydrophilic or hydrophobic groups or even by altering surface roughness. Our findings indicate that the untreated CH/PCL membrane demonstrated super-hydrophobic characteristics, as shown in Figure 3. To enhance the hydrophilicity of the electrospun surfaces, an alkali treatment method was employed. This treatment with NaOH successfully converted the surface characteristics to super-hydrophilic ones. Furthermore, all fiber mats augmented with silver nanoparticles (AgNPs) displayed contact angles of 0°, underscoring their super-hydrophilic nature.

**Figure 4:**
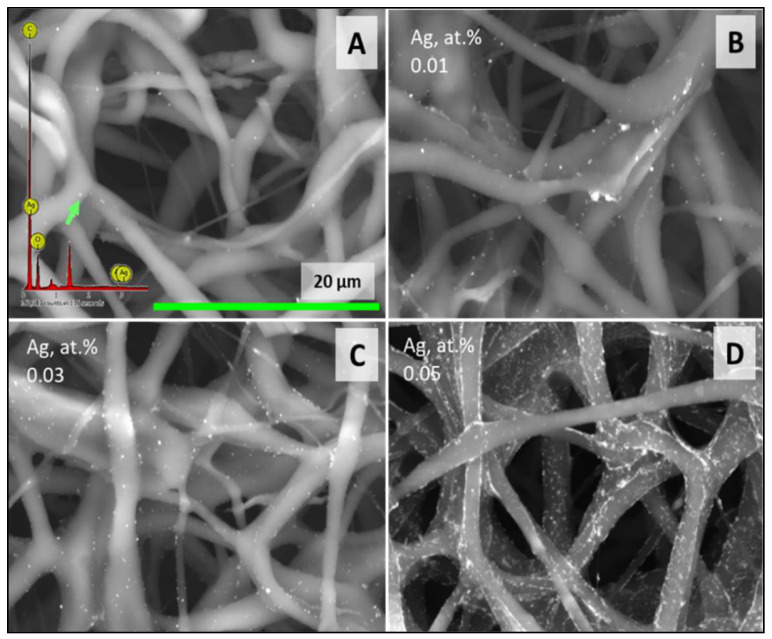
SEM image and EDX analysis of membranes loaded with 50 (**A**) (spot analysis), 100 (**B**), 200 (**C**), and 400 (**D**) µg (region analysis) of AgNPs.

The integration of AgNPs into the membrane did not adversely affect this surface property. The resultant hydrophilicity of the membranes laden with silver is anticipated to be advantageous for future cellular studies, given that scaffold wettability is crucial for protein adsorption and cell cultures. Higher magnification observations revealed that the membrane surfaces were uniformly coated with AgNPs, as confirmed by spot analysis, which verified the silver composition of these particles. A higher concentration of silver led to the formation of a thin particulate layer, particularly noticeable in the CH/PCL 200 AgNPs and CH/PCL 400 AgNPs samples. The average atomic concentration of silver became detectable by EDX analysis at concentrations beginning at 100 µg of AgNPs (Figure 4).

**Figure 5:**
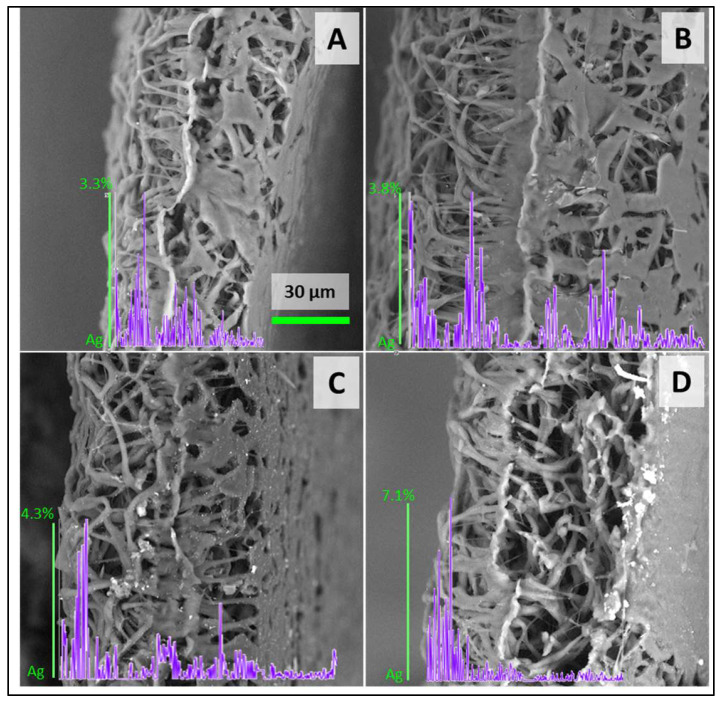
Scanning electron microscopy images of membranes’ cross-section with cross-sectional EDX line scan (violet diagrams) and EDS atomic % of Ag. Figure demonstrating membranes loaded with 50 (**A**), 100 (**B**), 200 (**C**), and 400 (**D**) µg of AgNPs.

The cross-sectional analysis demonstrated the intricate architecture of the membrane, featuring interconnected pores and the fibers’ smooth surfaces, as illustrated in Figure 5. EDX line scans substantiated the infiltration of silver within the membrane’s structure. Notably, the denser concentration of silver was predominantly localized in the upper layer of the membranes, suggesting a gradient in the distribution of the metallic nanoparticles.

### 3.2. Fourier Transform Infrared Spectroscopy (FTIR)

**Figure 6:**
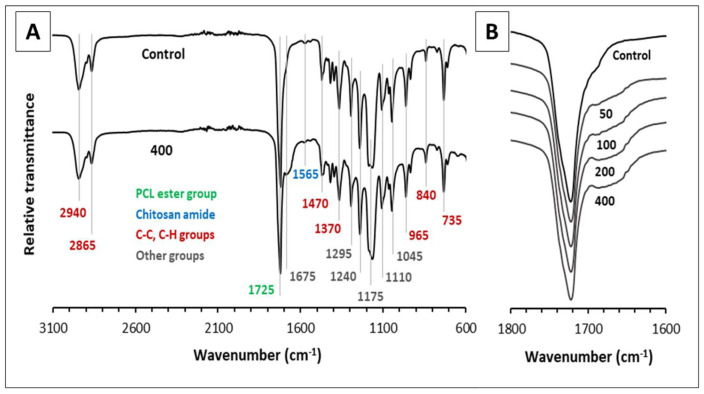
(**A**) FTIR spectra of the control sample and sample with the addition of 400 mg·L^−1^ of Ag, showing the most characteristic band positions with the accuracy ± 5 cm^−1^ in the wavelength range between 3100 and 600 cm^−1^; (**B**) FTIR spectra of all samples in the wavelength range between 1800 and 1600 cm^−1^.

FTIR spectroscopy analysis was used to characterize the phase composition of the samples by determining the functional groups, and the spectra captured are depicted in Figure 6. Regardless of the analyzed sample, in all cases, identical positions of the bands were revealed, while their shape and mutual intensity ratio turned out to have a highly repeatable manner, with a small exception for the spectrum region within 1800–1600 cm^−1^. In this regard, only the spectrum of the “control” sample and that with the highest amount of introduced silver were presented in Figure 6A, while Figure 6B was plotted to emphasize the sole difference arising from the silver concentration changes. Analyzing the spectra, an intense band at 1725 cm^−1^ stands out primarily, corresponding to the carbonyl C=O stretching vibration in esters [26]. Of all three organic components used to fabricate the nanofibers, the ester group is only contained in PCL, and hence, this band can be considered as reliable proof of its incorporation into the final product, and wherein in quite large amounts [27]. In turn, the structural feature of chitosan is determined by the presence of amide and amino groups, which are known to generate N–H bending vibrations, giving rise to many signals [28], and in particular, the one at 1570 cm^−1^, not overlapping with those produced by PCL and PEO [29,30]. However, this signal could be barely detected on both spectra presented in Figure 6A due to the fact that chitosan was used to prepare the nanofibers in relatively small amounts; however, it still is enough to prove the presence of this compound via FTIR spectroscopy analysis. Nevertheless, another band with similar intensity at 1640 cm^−1^, characteristic of the amide I group in chitosan, was not observed [28], which is probably due to its overlapping with the edge of the ester carbonyl band located nearby. The remaining bands that were visible on the spectra cannot be used for the speciation of phases since they represent the summing signals of two or three substances and are mainly included in the so-called “fingerprint” region, corresponding to the wavelength range from 600 to around 1500 cm^−1^ [31]. For example, chitosan and PEO are similar in terms of the presence of hydroxyl groups; chitosan and PCL both contain ether groups, while all three compounds have a branched carbon skeleton [27,31,32]. The latter is responsible for the appearance of bands at 2950 and 2860 cm^−1^ (C-H bond asymmetric and symmetric stretching) [27], the CH3 bending band at 1470 cm^−1^ [24], CH3 symmetrical deformations at 1375 cm^−1^ [26] out-of-plane CH bending at 965 cm^−1^ [27], as well as C-C stretching at 840 and 750 cm^−1^ [29]. The following group of bands arise from various modes of C-O and C-O-C bond stretching and are found at 1290, 1240, 1175, 1100, and 1045 cm^−1^ [27,28,30,32]. Among other things, it is worth noting that, in some cases, overlapping occurs, and the bands at 1375 and 1240 cm^−1^ are actually a sum of two separate signals. An additional contribution to the former is given by OH group bending [28], but the latter is intensified by CH_2_ group twisting [32]. Additionally, it should be noted that of all three organic components, only PCL has a unique signal in the “fingerprint” spectrum region with a maximum at 735 cm^−1^ [33], which is secondary evidence of its incorporation into the matrix of nanofibers. The addition of silver nanoparticles to the samples led to the appearance of an additional signal at the base of the C=O band with a weak maximum at ~1675 cm^−1^, and the intensity of this signal was found to correlate with the increase in silver concentration. These observations can be explained by the interaction of polymer substances with the surface of nanoparticles having a nature similar to the formation of hydrogen bonds, and also indicate the tangible embedding of particles into the structure of the nanocomposite [30]. Similar observations were reported in the research by Aframehr et al. for polyimide membranes containing nickel oxide particles [34]. Also, a scenario should not be excluded that the addition of silver could promote hydrogen bonding between polymers containing C=O groups and OH groups (e.g., PCL and PEO) [35]. Finally, the addition of silver slightly increased the transparency of the sample with respect to infrared radiation and uniformly reduced the intensity of the signals throughout the entire spectral range, which could be caused simply by a sample “dilution” effect and/or by partial absorption of infrared radiation by nanoparticles.

### 3.3. Silver Ions Release

**Figure 7:**
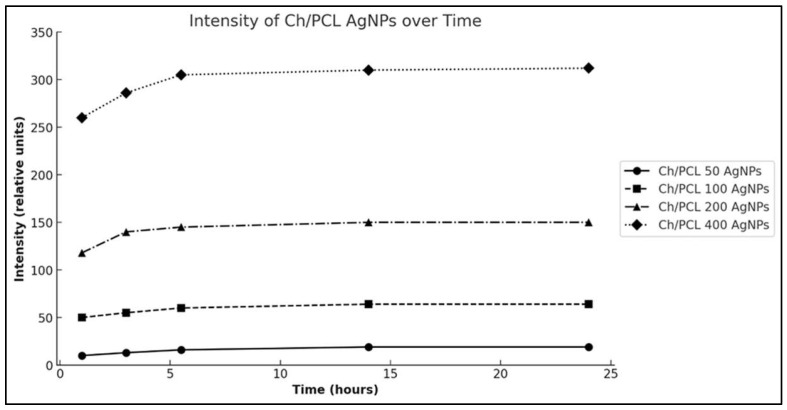
Comparison of the Ag ions release levels of CH/PCL-AgNPs membranes at each time point of the experiment.

The results of the experiments on the release of silver ions from nanofibers are depicted in Figure 7 and, according to the curve shapes, demonstrated typical dynamics characteristics for polymer matrices loaded with metal ions [36].

The rapid initial increase in the functions plotted and their subsequent emergence to a stable plateau indicates that within two hours, more than 90% of the silver was detached from the samples and also suggests that a significant portion of silver ions were already present in water within the first few seconds of the samples’ contact with water. Characteristically, the high similarity of the curves leads to the conclusion that the experiments are exceptionally repeatable and the progress of ion release is independent of the initial concentration of silver. Moreover, the ultimate amount of silver released showed a linear relationship with its total amount in the sample.

### 3.4. Biocompatibility Assessment

Previous studies have shown that sodium hydroxide treatment effectively increases the wettability of combined CH/PCL porous scaffolds, improving cell adhesion and proliferation [37]. Our preliminary studies confirm this tendency [38], as treating electrospun PCL membranes with an alkaline solution reduces the contact angle, facilitating the deposition and penetration of nanoparticles into the membrane thickness and promoting cell attachment and growth.

**Figure 8:**
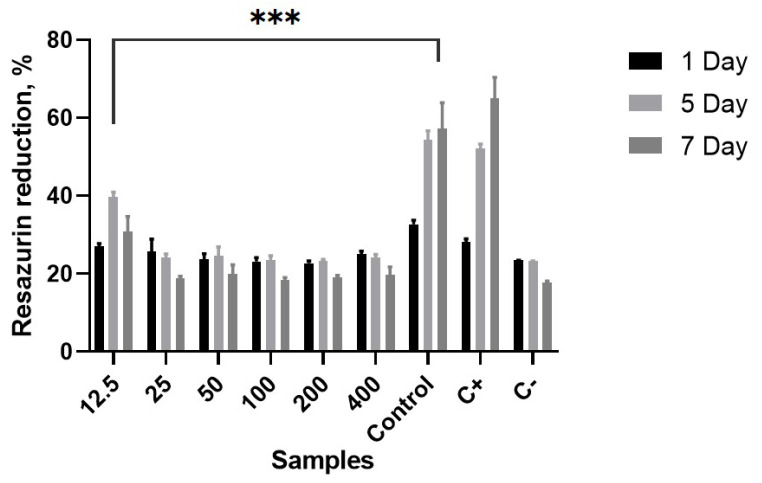
Resazurin reduction assay data on the proliferation of human keratinocytes during the 7-day experiment on different CH/PCL-AgNPs membranes (control—CH/PCL; numbers on samples mean amount of AgNPs µg/mL loaded to CH/PCL); *p*-value less than 0.001 flagged with three stars.

Figure 8 illustrates the cell attachment on membranes after 1-day culturing for all samples and demonstrates the proliferation of human keratinocytes for 1, 5, and 7 days for CH/PCL electrospun membrane. In turn, cell proliferation plays a crucial role in wound healing by allowing cells to multiply and create new extracellular matrix. During the 7-day investigation of CH/PCL nanocomposites with different AgNP concentrations, the sample loaded with 12.5 µg/mL demonstrated the best biocompatibility and least cytotoxicity compared to higher concentrations. The incorporation of silver nanoparticles into chitosan dressings should be investigated further to mitigate drug toxicity, particularly for wounds requiring immediate treatment. This exploration should consider both in vitro conditions and in vivo wound environments, taking into account the inherent differences between them [39].

### 3.5. Antibacterial Potential of Novel CH/PCL-AgNPs Membranes

**Figure 9:**
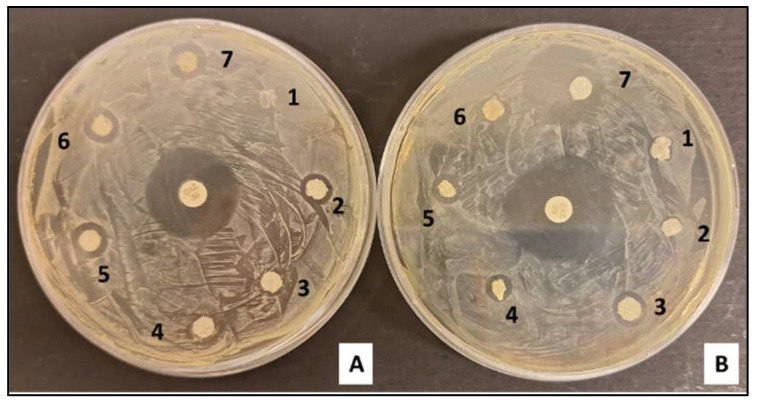
Disk-diffusion assay: zone of growth inhibition after *S. aureus* and *E. coli* (**A**) and (**B**) incubation with the electrospun membranes (CH/PCL control (1), CH/PCL—12.5 µg/mL (2), CH/PCL—25 µg/mL (3), CH/PCL—50 µg/mL (4), CH/PCL—100 µg/mL, (5) CH/PCL—200 µg/mL, (6) and (7) CH/PCL—400 µg/mL (7) AgNPs). Positive control was a disk with Ceftazidime (CAZ).

Our antimicrobial assays revealed that our electrospun samples’ bactericidal efficacy is contingent on the type of bacteria and the polymer composition utilized. The data, as depicted in Figure 9, demonstrate that fibers embedded with 400 µg/mL of silver nanoparticles (AgNPs) manifested pronounced antimicrobial activity against Escherichia coli, in stark contrast to the fibers lacking silver. However, in the case of Gram-positive Staphylococcus aureus, the antimicrobial differences remained marginal at nanoparticle concentrations of up to 50 µg/mL. It was observed that the inhibition zone was significantly larger for *E. coli* as opposed to *S. aureus*, particularly in membranes loaded with 100 µg/mL or more of AgNPs.

**Figure 10:**
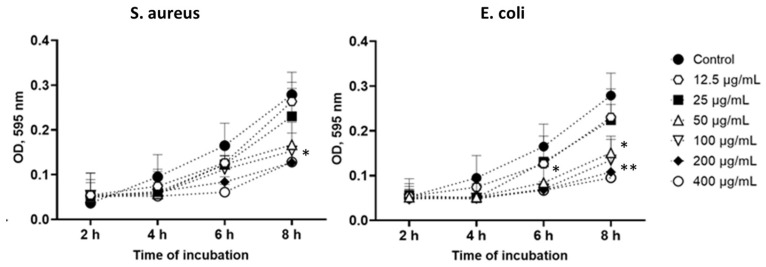
Evaluation of the antibacterial properties of CH/PCL-AgNPs membranes against *S. aureus* and *E. coli* (control—CH/PCL membrane; the amount of AgNPs loaded to CH/PCL represented in µg/mL); *—denotes significant differences between groups and control at *—*p* ≤ 0.05; **—*p* ≤ 0.01.

A significant AgNP dose-dependent reduction of *S. aureus* and *E. coli* bacterial population was observed up to the 6 h point of the assay (Figure 10). Moreover, it was noticeable that the time-dependent dynamics of the reduction rate of the total quantity of both bacterial strains were incubated with samples doped with 200 µg/mL and 400 µg/mL of silver nanoparticles. Otherwise, these silver-nucleated membranes had higher antimicrobial activity against Gram-negative bacteria than Gram-positive microorganisms after 2 h of the experiment. Chitosan has been proven to substantially affect Gram-negative bacteria more due to their higher hydrophilicity than Gram-positive bacteria [40]. As an outer membrane disruptor, chitosan possesses bacteriostatic properties rather than bactericidal. However, chitosan can be blended with other antimicrobial agents to increase biofunctional materials’ antimicrobial potential. Thereby, with up to 6 h of co-cultivation with membranes containing 50 µg/mL and 100 µg/mL of AgNPs, the reduction rate for *E. coli* growth was comparatively higher than for *S. aureus*. This approval of the other researchers’ results shows that silver doping into the nanofiber membranes provided a strengthening antibacterial effect on Gram-positive and Gram-negative bacteria [41]. The common mechanism of chitosan and silver nanoparticles’ antibacterial action was reinforced by the reported possible AgNP mechanisms, including membrane integrity alterations leading to intracellular leakage [42].

**Figure 11:**
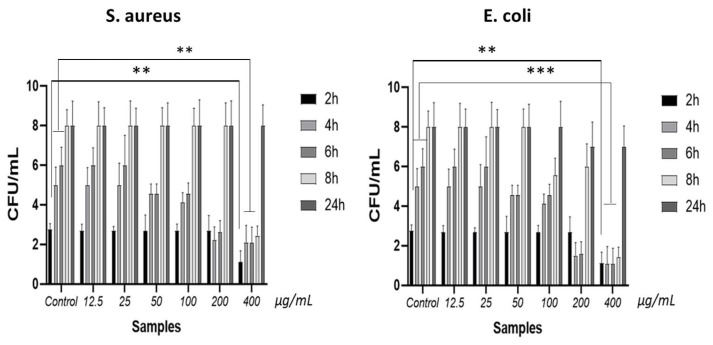
The count of living bacterial cells attached to the sample surfaces varied across different time intervals during the co-cultivation with *E. coli* and *S. aureus* (control—CH/PCL membrane; the amount of AgNPs µg/mL loaded to CH/PCL indicated on the horizontal axis); **—denotes significant differences between groups and control at **—*p* ≤ 0.01; ***—*p* ≤ 0.001.

The results of the biofilm formation survey revealed that the CH/PCL samples loaded with 200 µg/mL and 400 µg/mL of AgNPs display heightened anti-adhesive activity against both bacterial species for up to 8 h of incubation, as illustrated in Figure 11. Notably, at these specific time points, *E. coli* demonstrates a greater susceptibility to the antibacterial impact of the silver-loaded membranes. In particular, compared to the CH/PCL membranes without nanoparticles, the specimens infused with nanoparticles exhibit a more pronounced inhibition of bacterial biofilm growth after 2 h of co-cultivation with *S. aureus*. However, no significant difference is observed between the samples after 24 h of the experiment.

**Figure 12:**
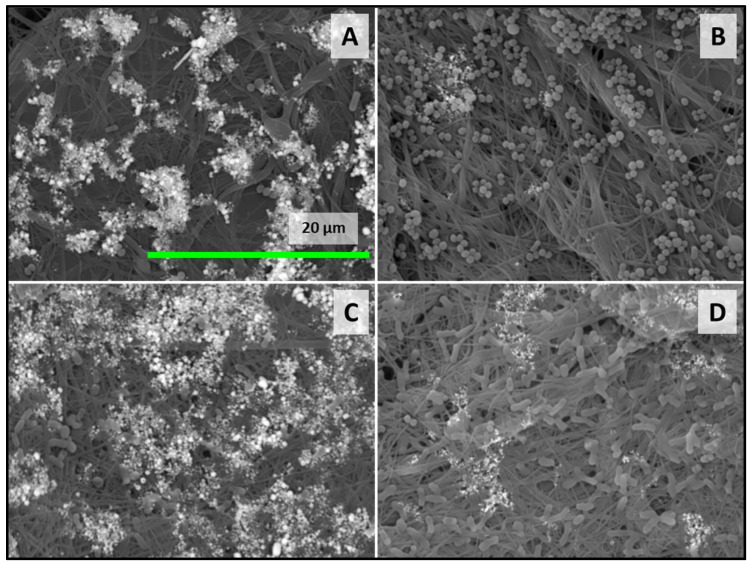
SEM images of the evaluation of the antibacterial properties of CH/PCL-AgNPs membranes against *S. aureus* loaded with 200 (**A**) and 25 (**B**) µg of AgNPs and *E. coli* loaded with 200 (**C**) and 25 (**D**) µg of AgNPs (presented the most representative SEM images).

The structure and size of fibers, along with the porosity of the membrane, play crucial roles in influencing bacterial proliferation. Research indicates that bacteria tend to adhere more readily to fibers with diameters comparable to their own size. In the electrospun membranes, the pore size was smaller than that of the microorganisms, effectively preventing bacterial infiltration and subsequent growth. Moreover, the shape of bacteria also influences adhesion, with round *S. aureus* showing greater adhesion and proliferation than the rod-shaped *E. coli*. The fibrous architecture of the membranes hampers the colonization process, leading to structural changes in rod-shaped bacteria and eventual cell death [43]. While the CH materials inherently possess antibacterial properties, studies demonstrate that the incorporation of AgNPs progressively enhances the antibacterial effectiveness with increasing exposure times. Furthermore, the antibacterial and antibiofilm activity assessments confirm the silver-loaded electrospun chitosan membranes’ ability to impede bacterial biofilm formation and exhibit strong antibacterial properties (Figure 12).

The time-dependent antibacterial activity of chitosan can be attributed to its degradation rate and the release of oligomers from the nanofibers. These oligomers facilitate the antimicrobial effect of chitosan by altering cell permeability, disrupting cell membranes, and penetrating cells, thereby inhibiting transcription. In addition to the aforementioned characteristics, the biodegradability and biocompatibility of chitosan further enhance its utility as an antimicrobial agent and drug delivery vehicle [44]. Thus, silver nanoparticles, loaded to CH/PCL membranes, starting at a concentration of 50 µg/mL, initially suppressed the growth of both microorganisms during the early stages of incubation but exhibited diminished effectiveness after 8 h. This observation correlates with the release profile of silver ions, confirming the early-stage efficacy of AgNPs. Some other previous researchers also confirmed the antibacterial properties of AgNPs [15,45,46].

Optimizing the level of silver nanoparticles (AgNPs) within dressings is crucial to ensure effective antimicrobial activity against microbial cells while maintaining safety for the eukaryotic cells within the wound bed. As repeatedly demonstrated, the dissolution of AgNPs into silver ions is the primary factor that contributes to toxicity in test systems. This suggests that the toxicity stems from ionic silver rather than the intrinsic properties of AgNPs [47]. Interpreting the in vitro assays requires caution, as the effects observed on individual parameters may not accurately reflect the interactions within the complex wound environment, which features intricate architecture in both the epidermis and dermis [48].

Wound exudate can bind excess silver ions, forming bio-inactive salts, which protect against metal toxicity [49]. In physiological salt concentrations, AgNPs tend to aggregate into larger formations, thereby mitigating the risk of small-sized particles translocating through the skin and slowing down their penetration into viable skin layers [50]. Additionally, studies have indicated that AgNPs from dressings predominantly remain localized in the wound area and do not penetrate deeper, as their primary action targets bacterial cells near the surface [51].

AgNP dressings speed up wound healing by regulating metalloproteinases, the crucial enzymes required for healing. Excessive metalloproteinases degrade essential components, but AgNPs control this, enhancing cell apoptosis and moderating inflammation. They also regulate TNF-α, preventing wound necrosis [52].

Therefore, based on our research findings, we anticipate that mechanisms deactivating AgNPs to a safe level will mitigate dose-dependent toxicity. Alternatively, impregnating the nanofibrous electrospun membranes with an appropriate amount of AgNPs can yield the necessary bacteriostatic effect to prevent inflammatory complications and support regeneration.

## 4. Conclusions

In this study, we successfully synthesized a novel CH/PCL-AgNPs membrane characterized by its high porosity and random fiber orientation that closely emulates the natural extracellular matrix. The membranes exhibited high structural and chemical integrity and a uniform distribution of AgNPs. The surface of the membrane was notably super-hydrophilic, an attribute that enhances its suitability for wound healing applications. This innovative material demonstrated remarkable biocompatibility, along with potent antibacterial activity against both Gram-positive and Gram-negative bacterial strains. Our findings indicate that CH/PCL composite membranes, crafted through a layer-by-layer electrospinning technique, hold significant promise for biomedical use, particularly as scaffolds for tissue engineering. The combined fabrication techniques employed to produce these composite materials, featuring AgNP-enhanced CH/PCL-AgNPs nanofibers, represent a novel approach to creating antibacterial biomaterials with superior efficacy. The synergy of high biocompatibility with antimicrobial potency thus positions our novel membranes as a vanguard in the field of regenerative medicine and wound care.

## Figures and Tables

**Table 1:** Summary of instrumental parameters for inductively coupled plasma mass spectrometry (ICP-MS).

Parameter	Setting
RF Power (W)	1550
Sampling Depth (mm)	8.0
Plasma Gas Flow Rate (L min^−1^)	15.0
Nebulizer Gas Flow Rate (mL min^−1^)	0.90
Makeup Gas Flow Rate (mL min^−1^)	0.0
He cell gas flow (mL min^−1^)	5.0
Extraction 1 Lens (V)	−17.2
Extraction 2 Lens (V)	−210
Omega Lens (V) 7.0	−200
Omega Bias Lens (V)	−130
Octopole Bias (V)	−18.0
Cell Gas Flow Rate (% of full scale)	20

## Data Availability

The raw data supporting the conclusions of this article will be made available by the authors on request.
